# Nitrogen-regulated antisense transcription in the adaptation to nitrogen deficiency in *Nostoc* sp. PCC 7120

**DOI:** 10.1093/pnasnexus/pgad187

**Published:** 2023-06-02

**Authors:** Manuel Brenes-Álvarez, Agustín Vioque, Alicia M Muro-Pastor

**Affiliations:** Instituto de Bioquímica Vegetal y Fotosíntesis, Consejo Superior de Investigaciones Científicas and Universidad de Sevilla, Américo Vespucio 49, 41092 Sevilla, Spain; Instituto de Bioquímica Vegetal y Fotosíntesis, Consejo Superior de Investigaciones Científicas and Universidad de Sevilla, Américo Vespucio 49, 41092 Sevilla, Spain; Instituto de Bioquímica Vegetal y Fotosíntesis, Consejo Superior de Investigaciones Científicas and Universidad de Sevilla, Américo Vespucio 49, 41092 Sevilla, Spain

**Keywords:** *Anabaena*, antisense RNA, citrate synthase, heterocyst, posttranscriptional regulation

## Abstract

Transcriptomic analyses using high-throughput methods have revealed abundant antisense transcription in bacteria. Antisense transcription is often due to the overlap of mRNAs with long 5′ or 3′ regions that extend beyond the coding sequence. In addition, antisense RNAs that do not contain any coding sequence are also observed. *Nostoc* sp. PCC 7120 is a filamentous cyanobacterium that, under nitrogen limitation, behaves as a multicellular organism with division of labor among two different cell types that depend on each other, the vegetative CO_2_-fixing cells and the nitrogen-fixing heterocysts. The differentiation of heterocysts depends on the global nitrogen regulator NtcA and requires the specific regulator HetR. To identify antisense RNAs potentially involved in heterocyst differentiation, we assembled the *Nostoc* transcriptome using RNA-seq analysis of cells subjected to nitrogen limitation (9 or 24 h after nitrogen removal) in combination with a genome-wide set of transcriptional start sites and a prediction of transcriptional terminators. Our analysis resulted in the definition of a transcriptional map that includes >4,000 transcripts, 65% of which contain regions in antisense orientation to other transcripts. In addition to overlapping mRNAs, we identified nitrogen-regulated noncoding antisense RNAs transcribed from NtcA- or HetR-dependent promoters. As an example of this last category, we further analyzed an antisense (*as_gltA*) of the gene-encoding citrate synthase and showed that transcription of as_*gltA* takes place specifically in heterocysts. Since the overexpression of *as_gltA* reduces citrate synthase activity, this antisense RNA could eventually contribute to the metabolic remodeling that occurs during the differentiation of vegetative cells into heterocysts.

**Significance Statement**

The transcription of bacterial genomes extends beyond the regions classically defined as genes. Antisense transcription of the complementary strand of coding regions is very frequently observed, not only because of an overlap between transcripts that are produced in adjacent genes, but also as autonomous, nonprotein-coding transcripts, whose expression is sometimes regulated in response to environmental changes. The consequences of antisense transcription are still a matter of debate, but accumulating evidence points to possible regulatory roles. In this context, the observation that antisense transcription takes place from nitrogen-regulated promoters controlled by the cyanobacterial regulators NtcA or HetR suggests that these antisense transcripts may play a role in adaptation to nitrogen stress and/or the differentiation of functional heterocysts.

## Introduction

Cyanobacteria are oxygen-producing photosynthetic organisms. In addition to light, which has a daily cycle of availability, nutrients that are critical for cyanobacterial growth, such as nitrogen, are also subject to fluctuations in natural, especially aquatic, environments, imposing the need for complex regulatory circuits to adapt quickly and accurately to changing conditions. Adaptation to nitrogen deficiency in both unicellular and filamentous cyanobacteria is under the control of the global nitrogen regulator NtcA (1) and involves the use of alternative nitrogen sources, as well as the recycling of cellular components (e.g. the phycobiliproteins) as a source of amino acids (2). In addition, some filamentous cyanobacteria can fix atmospheric nitrogen in specialized cells called heterocysts. Heterocysts differentiate from certain vegetative cells along the filaments in a process that involves structural and metabolic transformations to provide the microaerobic environment required for the function of nitrogenase, the enzyme that performs N_2_ fixation (3–5). Many of the gene expression changes associated with heterocyst differentiation are under the control of the HetR regulator (6). Genes whose expression is activated by HetR are induced specifically in cells that are becoming heterocysts. Genes exhibiting NtcA- or HetR-dependent expression constitute the NtcA and HetR regulons, respectively.

A previous genome-wide analysis of transcriptional start sites (TSSs) in *Nostoc* sp. PCC 7120 revealed two main categories of nitrogen-regulated transcripts (7). The DEF (deficiency) category includes transcripts that are similarly regulated in the wild-type (WT) and *hetR* mutants, which cannot differentiate heterocysts. Although, in some cases, NtcA might be indirectly responsible for the observed regulation of DEF genes, most promoters in this category contain NtcA-binding sites, and therefore, the corresponding genes constitute the directly regulated NtcA regulon. The second category of nitrogen-regulated promoters, the DIF (differentiation) category, includes transcripts that require HetR for induction and, therefore, are specifically related to heterocyst differentiation. Two groups of genes can be defined in this category (E-DIF and L-DIF) based on their temporal pattern of induction during the early (E) or late (L) stages of differentiation, respectively (6). Most genes in the E-DIF category are transcribed from promoters with conserved sequence motifs (DIF1 or DIF2 motifs), associated with their expression specifically in cells that are initiating differentiation as heterocysts (6, 7).

Previous transcriptomic analyses carried out in both unicellular and filamentous cyanobacteria have shown abundant transcription of antisense RNAs (asRNAs) (8). In the case of *Nostoc* sp. PCC 7120, ∼30% of TSSs determined using differential RNA-seq (dRNA-seq) (7) were assigned to the antisense category (aTSS), consistent with the abundance of antisense transcripts in RNA-seq data sets (9). In previous work, we have characterized two antisense RNAs with heterocyst-specific transcription, *as_glpX* (10) and NsiR1 (11). Here, we aim at the global analysis of nitrogen-regulated antisense transcription in *Nostoc*, with a focus on asRNAs whose expression is restricted to heterocysts. The identification of asRNAs that are under control of the regulators involved in heterocyst differentiation, and specifically transcribed during the transformation of a vegetative cell into a mature heterocyst, points to a participation of these asRNAs in the posttranscriptional regulation of this unique cell differentiation process.

## Results

### Nitrogen-regulated transcriptome in *Nostoc* sp. PCC 7120

We sequenced the transcriptome of *Nostoc* cells growing in the presence of ammonium (time 0) or subjected to nitrogen deprivation for 9 or 24 h (time 9 and time 24), two time points suitable for the analysis of transcriptional changes associated with the early and late stages of heterocyst differentiation, respectively. RNA from two biological replicas of each condition was sequenced to a depth of 17–21 million paired-end reads each sample. A transcriptome was assembled as detailed in Materials and Methods in combination with a previously available data set of transcription start sites obtained by dRNA-seq (7) and a prediction of transcription terminators. 4,039 transcripts were predicted, 3,127 of them associated with TSS previously defined using cells grown in the presence of ammonium or after 8 h of combined nitrogen deprivation (7) (Data Set S1).

Data Set S1 contains comparisons of the expression of each transcriptional unit (TU) at 9 or 24 h after nitrogen removal versus time 0. Consistent with previous analysis of the response of *Nostoc* sp. PCC 7120 to nitrogen deficiency, transcripts corresponding to NtcA-regulated genes in the DEF category (e.g. *nrrA*, *ntcB*) (7), or HetR-dependent, heterocyst-specific genes in the DIF category (e.g. *het*, *dev*, *nif* genes) (7) are among the transcripts induced more than eight-fold after 9 h of nitrogen starvation [i.e. log_2_ (FC 9–0) > 3]. Also, in agreement with previous transcriptomic studies (6), genes expressed during early stages of heterocyst differentiation (such as *hep* genes involved in polysaccharide deposition) show higher expression at 9 h than at 24 h after nitrogen removal. In contrast, genes whose expression occurs in mature heterocysts (such as *nif* genes that encode nitrogenase and associated functions) show higher expression at 24 h than at 9 h after nitrogen removal.

It should be noted that although this transcriptome was assembled on the basis of RNA from cells growing under only 3 different conditions, the assembled transcriptome contains transcripts covering 75% of all *Nostoc* sp. PCC 7120 genes (4,614 genes of the total of 6,137 genes annotated for this organism according to the NCBI annotation). Therefore, this transcriptome may represent a good overview of the overall transcriptome of our organism, including housekeeping genes expressed independently of environmental condition. We have analyzed some global parameters of the assembled transcriptome, such as the length distribution of transcripts, 5′UTRs and 3′UTRs (Fig. S1). One of the longest transcripts corresponds to the operon enconding ribosomal proteins. Also some operons essential for heterocyst differentiation, such as the *hep* operon or the *hgl* operon, are among the longest TUs in the *Nostoc* transcriptome (Data Set S1). The *Nostoc* transcriptome also contains long 5′UTRs and 3′UTRs (Fig. S1B and C). The presence of long 3′UTRs could be explained by the absence of Rho-termination factors in cyanobacteria (12, 13). To test this hypothesis, we compared the length distribution of 3′UTRs of *Nostoc* and *Synechocystis* sp. PCC 6803, a unicellular cyanobacterium, with the length of 3′UTRs in two gram-negative bacteria enconding homologs of Rho termination factor, *Campylobacter jejuni* 81116 (14) and *Helycobacter pylori* 26695 (15, 16), for which available transcriptomic data allow a similar analysis (17). According to these comparisons, significantly longer 3′UTRs were observed in the two tested cyanobacterial transcriptomes than in the other two transcriptomes analyzed (Fig. S1C). However, the most striking feature was the significant increase in the length of 5′UTRs in the cyanobacterial transcriptomes (Fig. S1B). In all four organisms analyzed, their transcriptomes showed a preference for 5′UTRs with an average length of 30 nucleotides. Because long 5′UTR can be involved in regulation, we also compared the length of TUs corresponding to previously identified HetR-dependent genes (6) with the rest of TUs in the data set. In fact, the length of 5′UTRs in HetR-regulated transcripts is significantly higher than in non-HetR-regulated transcripts (Fig. S2).

### Nitrogen-regulated antisense transcription in *Nostoc* sp. PCC 7120

The presence of long 5′UTRs and 3′UTRs in the *Nostoc* transcriptome led us to analyze the possible antisense regulation exerted by overlapping protein-coding mRNAs during the response to nitrogen deprivation. All TUs that overlapped with a TU of the other strand for >50 nucleotides were classified as asRNAs (Data Set S1). These represent 2,620 transcripts (65%), which is consistent with the prevalence of antisense transcription previously described for cyanobacteria (8, 18). Most asRNAs were, in fact, protein-coding mRNAs that overlapped (1757). However, we also found 863 antisense transcripts that were not predicted to encode any protein.

Among the TUs classified as asRNAs, we selected for further analysis those showing log_2_ (FC 9-0) above 3. In 23 cases, the asRNA is an mRNA containing at least one coding sequence (Table 1). Among these nitrogen-regulated overlapping mRNAs, we found genes whose expression depends on HetR or NtcA. Eight mRNAs include genes that belong to one of the two previously described groups of HetR-dependent transcripts (named E-DIF and L-DIF according to their early or late expression, respectively) (6). Most overlapping mRNAs in the E-DIF group, such as those of *alr4984*, *all2571*, *alr3195,* or *alr3676*, are transcribed from promoters bearing a DIF1 motif associated with heterocyst-specific expression (6, 7). In some other cases, nitrogen-regulated overlapping mRNAs are transcribed from promoters containing NtcA-binding sites, such as those of *asl2052*, *nrrA*, or *all1395* (7). We noticed a prevalence of tail-to-tail disposition, but there are also examples of head-to-head disposition. As an illustration of tail-to-tail or head-to-head disposition, two examples involving antisense HetR-dependent TUs (TU03408, *as_susA* and TU00968, *as_all1549*-*patD*) are shown in Fig. S3 together with TU01524, previously described *as_fraE* (TU01524), which corresponds to overlapping transcription of the 3′UTR of *fraF* mRNA (19) but with log_2_ (FC 9-0) = 1.95 falls below the threshold log_2_ (FC 9-0) > 3 applied above.

**Table 1.** Selected nitrogen-regulated mRNAs antisense to another mRNA.

Nitrogen-regulated transcript					Overlapped transcript	
TU	log_2_ (FC 9-0)	log_2_ (FC 24-0)	Gene(s)	Regulation^a^	TU	Gene(s)
TU01301	7.28	6.04	*asl2052*	NtcA	TU01300	*ggt*
TU03408	6.23	4.75	*alr4984*	E-DIF	TU03409	*susA*
TU02503	6.18	3.76	*all3772*	E-DIF	TU02502	*alr3771*
TU00203	5.42	7.24	*hesF*	L-DIF	TU00204	*prfB to all0268*
TU00037	5.27	3.50	*all0059*		TU00036	*alr0056–alr0058*
TU02896	5.15	3.59	*nrrA*	NtcA	TU02895	*alr4310*
TU01911	4.82	5.80	*all2965*	L-DIF	TU01912	*alr2966–alr2967*
TU01656	4.65	2.96	*all2571*	E-DIF	TU01655	*petR-petP*
TU00909	4.56	5.50	*nifV1 to alr1410*		TU00910	*asl1412–all1411*
TU01443	4.33	4.49	*asl2299*		TU01442	*alr2298*
TU00901	4.30	4.47	*all1395–asl1394*	NtcA	TU00899	*alr1390 to PCC7120DELTA_RS32405*
TU02336	4.01	3.59	*alr3554*		TU02337	*all3556-ilvB*
TU02841	3.86	2.58	*PCC7120DELTA_RS22895*		TU02840	*argJ2*
TU02078	3.81	2.08	*alr3195*	E-DIF	TU02079	*asl3196*
					TU02080	*all3197*
TU01767	3.78	3.22	*all2747*		TU01765	*alr2745–alr2746*
TU02125	3.75	2.59	*asr3279*	NtcA	TU02124	*all3278*
TU01093	3.71	2.86	*asr1734*		TU01094	*all1736*
TU00968	3.64	2.72	*patD*		TU00967	*all1549*
TU02430	3.59	1.80	*alr3676*	E-DIF	TU02429	*all3675*
					TU02431	*all3677*
TU00398	3.43	2.74	*alr0627*		TU00399	*asl0628*
TU01671	3.39	2.88	*alr2582*	L-DIF	TU01673	*fecC1 to fecE1*
			*fecB1*			
TU03256	3.23	2.22	*alr4800*		TU03257	*rpsF-all4801*
TU01496	3.17	1.96	*ald*		TU01497	*all2358 to phnE2*

a NtcA indicates that the nitrogen-regulated gene is preceded by a previously described NtcA-regulated promoter (7, 60). E-DIF or L-DIF indicates that the nitrogen-regulated gene appears in one of the previously defined groups of HetR-dependent genes (early or late, respectively) (6).

In contrast to the above-described overlapping mRNAs, in 12 cases, antisense transcripts with log_2_ (FC 9-0) > 3 correspond to TUs that do not contain any annotated coding sequence. These noncoding antisense transcripts are produced from TSSs annotated as antisense TSS (aTSS) or nTSS (noncoding TSS) (7) (Table 2). Transcription from some of these TSS is driven by promoters bearing DIF1 motifs (*as_gltA*, *as_hglD*, *as_acsF*) or NtcA-binding sites (*as_pstS2*, *as_pknC*, *as_fdxB*, *as_leuA*) (7). All three asRNAs transcribed from DIF1-containing promoters belong to the E-DIF group of HetR-dependent transcripts (6). Two nitrogen-regulated noncoding asRNAs previously described appeared in our data set with log_2_ (FC 9-0) below 3. Data for the regions corresponding to *as_glpX* [TU00652; log_2_ (FC 9-0) = 2.27] (10) and to *as_anacyp40* [TU03461; log_2_ (FC 9-0) = 1.98] (6, 20), both transcribed from DIF1-containing HetR-dependent promoters, are shown in Fig. S4.

**Table 2.** Nitrogen-regulated noncoding RNAs antisense to an mRNA.

Nitrogen-regulated transcript					Overlapped transcript	
TU	Name	Log_2_ (FC 9-0)	Log_2_ (FC 24-0)	Regulation^a^	TU	Gene(s)
TU02147		5.11	5.09		TU02146	*all3305*
TU03089	*as_pstS2*	4.51	3.60	NtcA	TU03088	*pstS2-pstC2-pstA2-pstB2*
TU03269	*as_pknC*	4.43	4.50	NtcA	TU03268	*pknC*
TU00166	*as_gltA*	4.43	3.70	E-DIF	TU00167	*gltA*
TU03638	*as_hglD*	4.40	3.42	E-DIF	TU03636	*asr5350-hglEF-hglG-hglD-hglC-hglA-hglB*
TU00092		4.25	4.33	L-DIF	TU00091	*alr0130*
TU01725		4.05	3.53		TU01724	*all2700*
TU01622	*as_fdxB*	4.03	3.42	(NtcA)	TU01621	*fdxB*
TU02297	*as_leuA*	3.79	2.87	NtcA	TU02296	*leuA*
TU01075		3.79	3.48		TU01074	*alr1712*
					TU01076	*alr1713-asr1714-alr1715*
TU00785		3.56	2.96		TU00784	*alr1240-alr1241-alr1242*
TU00867	*as_acsF*	3.02	1.97	E-DIF	TU00866	*acsF*

a NtcA indicates that the nitrogen-regulated gene is preceded by a previously described NtcA-regulated promoter (7) or, in the case of TU01622, a promoter with an NtcA-binding site identified by ocular inspection (NtcA). E-DIF or L-DIF indicates that the nitrogen-regulated gene appears in one of the previously defined groups of HetR-dependent genes (early or late, respectively) (6).

Taken together, these observations suggest that both the NtcA-regulated responses (not specifically linked to heterocyst differentiation) and the HetR-regulated responses (specifically associated with heterocyst differentiation) include abundant transcription of asRNAs, either because of overlap between mRNAs located in a head-to-head or tail-to-tail disposition or due to the transcription of *bona fide*, noncoding asRNAs.

### Noncoding antisense transcripts in the NtcA and HetR regulons

Observed overlap of antisense protein-encoding mRNAs with long 3′UTRs could be the result of improper transcription termination. However, the presence of noncoding antisense RNAs with strict transcriptional regulation strongly suggests a regulatory function for this type of RNAs. We have selected for further validation nitrogen-regulated TU with log_2_ (FC 9-0) > 3 that fall in the category of noncoding asRNAs (i.e. are not mRNAs) and are transcribed antisense to genes with a previous annotation (*as*_*pstS2*, *as*_*hglD*, *as*_*fdxB*, *as*_*gltA, as_acsF*, *as_leuA*, *as_pknC*; Table 2). These asRNAs were transcribed antisense to genes that belong to different functional categories, from amino acid biosynthesis (*leuA*) to regulation (*pknC*, protein kinase).

**Fig. 1.**
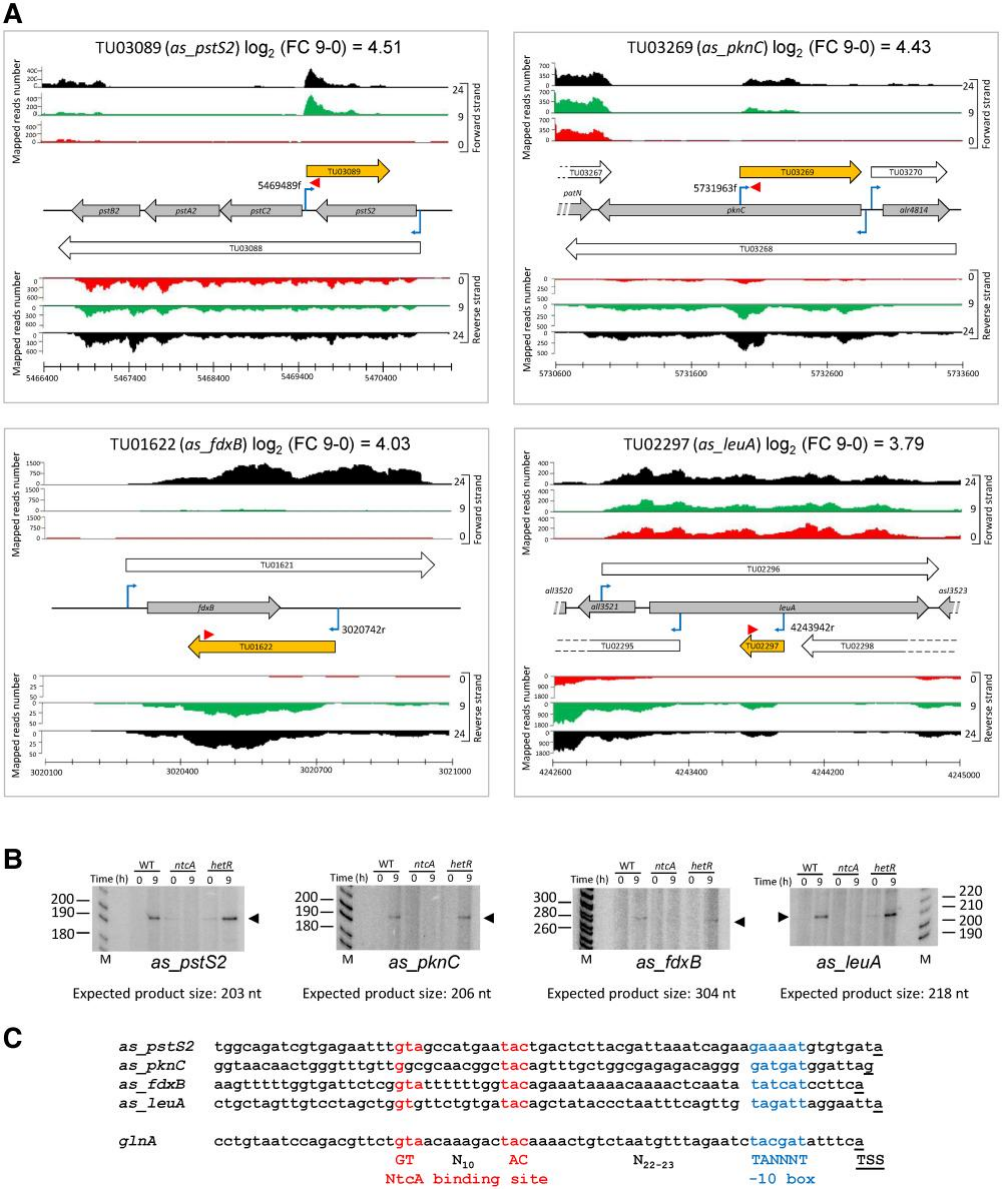
Examples of NtcA-dependent nitrogen-regulated asRNAs. A) Distribution of reads mapped in relevant regions of RNA obtained from cells grown in the presence of ${\mathrm{NH}}_{4}^{+}$ (red), or after 9 (green) or 24 h (black) in the absence of combined nitrogen. Annotated ORFs are represented by gray arrows. TUs are represented by white arrows with their corresponding identification. Nitrogen-regulated antisense TUs are colored orange. Previously identified TSSs are indicated by bent blue arrows. Genomic coordinates denote the position on the *Nostoc* sp. PCC 7120 chromosome. The scale indicates the number of mapped reads per nucleotide position. B) Primer extension analysis of the asRNAs shown in A using RNA extracted from the cells of WT, *ntc*A mutant, or *hetR* mutant grown in the presence of ${\mathrm{NH}}_{4}^{+}$ (0), or after 9 h (9) in the absence of combined nitrogen. The positions of the primers used are indicated by red triangles in A. Black triangles point to the primer extension products. The expected sizes of the extension products are included under each panel. Relevant size markers (M) are indicated. C) Sequences upstream of the TSS of the four analyzed asRNAs, compared with the sequence upstream of the NtcA-regulated TSS of *glnA* and the consensus for NtcA-activated promoters. The conserved NtcA-binding motif is colored red. The −10 box is highlighted in blue, and the TSS is underlined.

Figure 1A shows RNA-seq data for four selected asRNAs transcribed from NtcA-regulated promoters, after 9 or 24 h of nitrogen deprivation. Their transcription was verified by primer extension using oligonucleotides located at least 100 nucleotides downstream of the associated aTSS (7) (see Materials and Methods for details). Products of the expected size (from the position of the oligonucleotide used in the primer extension reaction to the position of the aTSS) were observed (Fig. 1B). In the four cases, the expression of inducible asRNAs did not take place in the *ntcA* mutant strain, but was still observed in the *hetR* mutant strain, confirming that these asRNAs belong to the NtcA regulon and the DEF category, according to (7). Comparison of promoters upstream of the corresponding aTSS shows the presence of putative NtcA-binding sites in a position compatible with direct activation by NtcA (the well-described NtcA-regulated promoter of *glnA* is included for comparison; Fig. 1C).

**Fig. 2.**
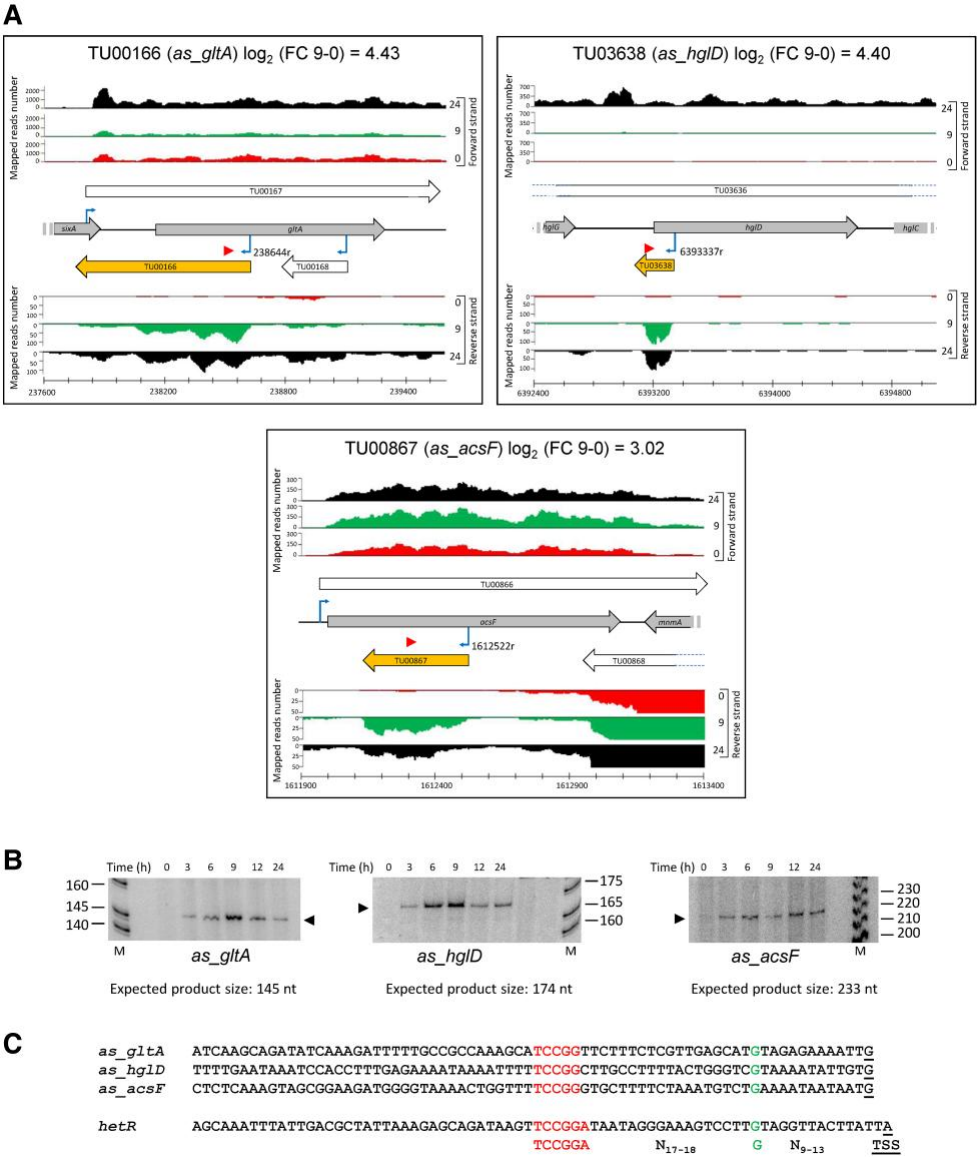
Examples of HetR-dependent nitrogen-regulated asRNAs. A) Distribution of reads mapped in relevant regions of RNA obtained from cells grown in the presence of ${\mathrm{NH}}_{4}^{+}$ (red), or after 9 (green) or 24 h (black) in the absence of combined nitrogen. All labels are as in Fig. 1. For *as_acsF*, the scale of the reverse strand was adjusted for better visualization of the reads mapped to the antisense RNA. B) Primer extension analysis of asRNAs shown in A using RNA extracted from cells of WT strain grown in the presence of ${\mathrm{NH}}_{4}^{+}$ (0), or after 3 to 24 h in the absence of combined nitrogen. The positions of the primers used are indicated by red triangles in A. Black triangles point to the primer extension products. The expected sizes of the extension products are included under each panel. Relevant size markers (M) are indicated. C) Sequences upstream of the TSS of the three analyzed asRNAs compared with the sequence upstream of the heterocyst-specific TSS of *hetR*. The conserved DIF1 motif is highlighted in red. A conserved G is highlighted in green, and the TSS is underlined.

Figure 2A shows RNA-seq data of three selected asRNAs associated with aTSS in the DIF category, therefore potentially linked to heterocyst differentiation (*as_hglD*, *as_gltA*, and *as_acsF*). Again, we verified nitrogen-regulated transcription from the corresponding aTSS along a time course of nitrogen deprivation by primer extension, obtaining products of the expected sizes (Fig. 2B). Consistent with their classification as E-DIF (6), expression of all three was already induced 3 h after nitrogen removal. In the three cases, the comparison of the promoters upstream of the corresponding HetR-dependent aTSS (7) reveals the presence of DIF1 motifs, associated with heterocyst-specific expression (the DIF1-containing promoter of *hetR* is included for comparison; Fig. 2C).

### A heterocyst-specific asRNA in the *gltA* gene

**Fig. 3.**
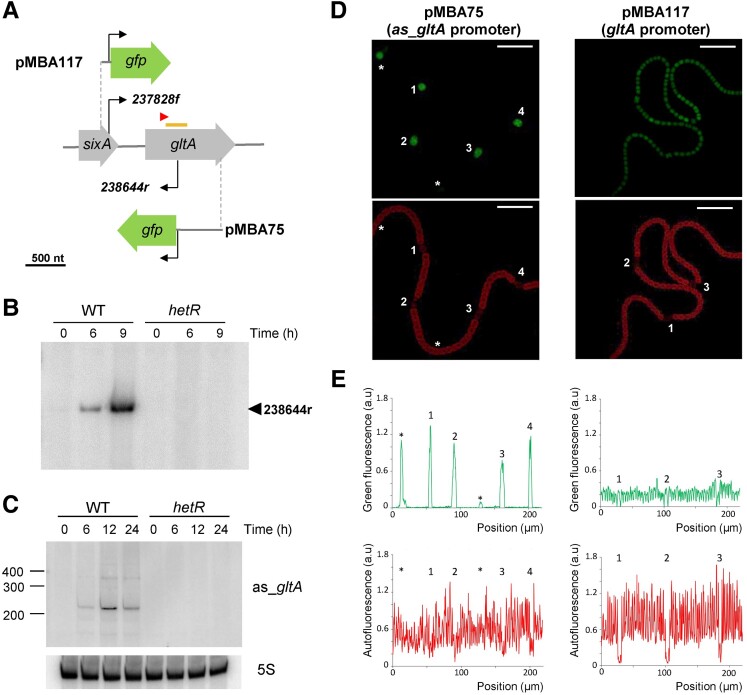
Expression pattern of the *gltA* and *as_gltA* promoters in *Nostoc* filaments. A) Scheme of the promoter fusions to GFP in plasmids pMBA75 and pMBA117. B) Primer extension analysis of *as_gltA* using RNA extracted from cells of WT or *hetR* mutant grown in the presence of ${\mathrm{NH}}_{4}^{+}$ (0), or after 9 h in the absence of combined nitrogen. C) Northern blot of *as_gltA* with RNA extracted from cells of WT or *hetR* mutant grown in the presence of ${\mathrm{NH}}_{4}^{+}$ (0), or after 6 to 24 h in the absence of combined nitrogen. As a loading control, the filter was hybridized to a probe for 5S RNA. D) Representative confocal fluorescence images of *Nostoc* filaments carrying the *gfp* gene under the control of the *as_gltA* promoter (plasmid pMBA75, left) or the *gltA* promoter (plasmid pMBA117, right) and growing on top of medium lacking any source of combined nitrogen are shown for the green channel (GFP fluorescence) and the red channel (autofluorescence). Numbers indicate heterocysts, and asterisks indicate immature heterocysts. Scale bars 20 µm. E) Quantification of the signals for the green and red channels in the images shown in D. Left, pMBA75; right, pMBA117. Numbers and asterisks as in D.

One of the nitrogen-regulated noncoding asRNAs verified above, *as_gltA*, TU00166, is transcribed from a TSS at position 238644r, on the antisense strand of *gltA*, encoding citrate synthase (Fig. 3). This TSS is located at position +474 relative to the start codon of *gltA* (Fig. 3A). The accumulation of the corresponding transcript is induced in a HetR-dependent manner, as demonstrated by primer extension (Fig. 3B). The 3′ end of *as_gltA* was identified by 3′RACE. The most abundant sequenced products ended between positions 238399 and 238424, but less abundant, longer products extending to position 238278 were also detected (not shown). The accumulation of *as_gltA* was verified by Northern blot, which revealed, in agreement with the RACE results, an abundant band slightly above 200 nt and another less abundant band, above 300 nt, as well as some signals above the 400 nt size marker. No expression of the *as_gltA* RNA was observed in the *hetR* mutant (Fig. 3B and C). The size of the predicted transcript TU00166 (866 bp) suggests that the major band detected by Northern blot might be generated by processing TU00166.

The presence of a DIF1 motif-containing promoter (Fig. 2) and the HetR dependence of *as_gltA* transcription (Fig. 3B and C) suggest that the expression of *as_gltA* is heterocyst specific. To confirm this hypothesis, we constructed a *Nostoc* strain that carries the *gfpmut2* gene under control of the *as_gltA* promoter (positions 238804 to 238632, sequence −160 to +13 with respect to the TSS; see pMBA75 in Fig. 3A). We also constructed a strain that carries the *gfpmut2* gene under the control of the *gltA* promoter (positions 237627 to 237874, sequence −200 to +47 with respect to TSS; see pMBA117 in Fig. 3A). GFP expression was analyzed by confocal fluorescence microscopy in both strains (Fig. 3D). Quantification of GFP fluorescence along the filament demonstrated that transcription from the promoter of *as_gltA* took place specifically in heterocysts and could be detected in cells that had only initiated the process of differentiation, even before characteristic signs of differentiation such as reduced red autofluorescence or increased size were observed (see cells indicated with asterisks in the left panels of Fig. 3D and E). Early expression of *as_gltA* in cells that are only initiating differentiation as heterocysts is consistent with the expression pattern observed for genes that contain DIF1-containing promoters such as NsiR1 (21) or *hetR* (6). In contrast, there was a similar level of GFP expression from the *gltA* promoter along the filament in both vegetative cells and heterocysts (right panels in Fig. 3D and E).

### Effect of *as_gltA* on the expression of citrate synthase

**Fig. 4.**
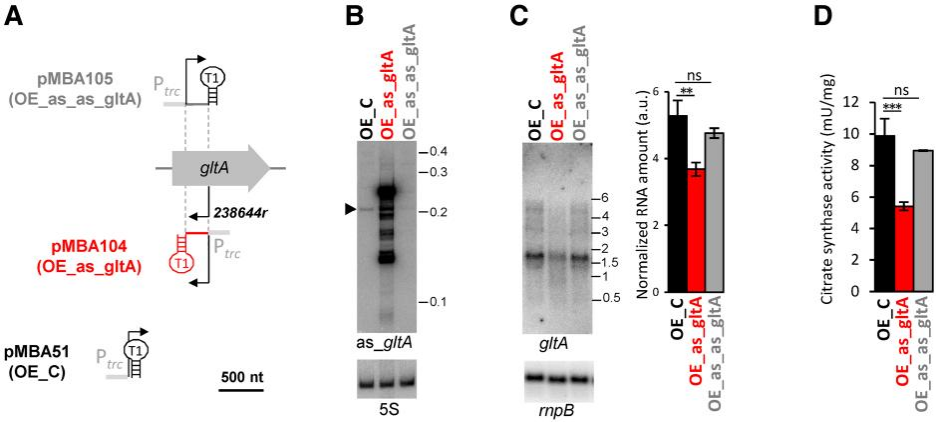
Effect of *as_gltA* overexpression or depletion on *gltA* expression and citrate synthase activity. A) Scheme of the DNA fragments cloned in the plasmids constructed to generate *Nostoc* strains with altered levels of *as_gltA* RNA. Transcriptional start sites (bent arrows), T1 terminators (stem loops), and *trc* promoters are indicated. B) Northern blot analysis of the accumulation of *as_gltA* transcript using RNA extracted 18 h after nitrogen removal from the control strain (OE_C), the strain overexpressing *as_gltA* (OE_as_gltA), and the strain depleted of *as_gltA* (OE_as_as_gltA). 5S RNA was used as a loading control. C) Left, Northern blot analysis of the accumulation of *gltA* transcript using RNA extracted 18 h after nitrogen removal from the control strain (OE_C), the strain overexpressing *as_gltA* (OE_as_gltA), and the strain depleted of *as_gltA* (OE_as_as_gltA). *rnpB* was used as a loading control. Right, quantification of *gltA* mRNA in the three strains. Average and standard deviation of the quantification of three biological replicates. D) Citrate synthase activity in crude extracts prepared 18 h after nitrogen removal from the control strain (OE_C), the strain overexpressing *as_gltA* (OE_as_gltA), and the strain depleted of *as_gltA* (OE_as_as_gltA). Average and standard deviation of the quantification of three biological replicates (ns, not significative; **P < 0.01; ***P < 0.001, Student's t test).

We hypothesize that transcription of *as_gltA* might regulate the accumulation or translation of *gltA* mRNA and, therefore, the amount of citrate synthase. Because *as_gltA* is transcribed specifically in heterocysts (Fig. 3D and E), any effect on the accumulation of *gltA* would be restricted to these cells, which represent a small percentage of the cells in the filaments. Therefore, to analyze possible effects of *as_gltA*, we have constructed a strain of *Nostoc* that overexpresses *as_gltA* from a constitutive promoter in all cells of the filament (OE_as_gltA) and a strain that overexpresses an antisense RNA of *as_gltA* (OE_as_as_gltA). The 245 nt fragment defined as the main product of *as_gltA* transcription by Northern blot (Fig. 3C) and 3′RACE (positions 238644r to 238399r) was cloned between the *trc* promoter and the strong T1 transcriptional terminator of *rrnB* of *Escherichia coli*, and the resulting plasmid (pMBA104) was introduced in *Nostoc*, generating strain OE_as_gltA. To remove *as_gltA* without altering its genomic region, we cloned the same fragment in reverse orientation downstream of the *trc* promoter to generate a perfect antisense that would act as a sponge, neutralizing *as_gltA*. The resulting plasmid (pMBA105) was introduced in *Nostoc*, generating strain OE_as_as_gltA. We have previously used this strategy to deplete different transcripts in *Nostoc* (22, 23). pMBA51, a plasmid that does not have an insert between the *trc* promoter and the T1 terminator (10), was also introduced in *Nostoc* as a control, generating strain OE_C (Fig. 4A). We first analyzed the expression of *as_gltA* by Northern blot in the three strains after 18 h of culture in medium lacking combined nitrogen (Fig. 4B). As expected, strain OE_as_gltA accumulated a much higher amount of *as_gltA* than the control strain OE_C, while strain OE_as_as_gltA, which expresses an antisense RNA to *as_gltA*, had a reduced amount of as_*gltA*. No significant growth defect was detected in strains OE_as_gltA and OE_as_as_gltA after combined nitrogen depletion (not shown). We next studied the effect of the changes in the amount of *as_gltA* on the accumulation of *gltA* mRNA by Northern blot (Fig. 4C). Overexpression of *as_gltA* resulted in a 30% reduction in the *gltA* mRNA signal with respect to the control strain, while depletion of *as_gltA* had no significative effect. Finally, we measured citrate synthase activity in the three strains (Fig. 4D). Overexpression of *as_gltA* resulted in a 45% reduction in citrate synthase activity, while depletion of *as_gltA* had no significative effect.

## Discussion

Photosynthetic microorganisms face unique challenges that make their survival dependent on sophisticated regulatory circuits. In the case of heterocystous cyanobacteria, adaptation to nitrogen limitation involves a cell differentiation process that completely transforms the physiology of the filament as a whole to survive using atmospheric N_2_ as the only nitrogen source. In this context, regulatory mechanisms orchestrated by noncoding RNAs have already been described that participate in the differentiation of certain cells into heterocysts. Differential accumulation in heterocysts of trans-acting sRNAs NsiR1, NsiR3, or NsiR4 modulates the differentiation process (11, 23) or the activities of several enzymes (24, 25), specifically in developing heterocysts. In this work, we provide the first transcriptional map of a heterocystous cyanobacterium along the heterocyst differentiation process. The boundaries of the transcripts are based on conventional RNA-seq data from this work, an available TSS data set (7), and a prediction of transcriptional terminators.

Antisense transcription is widespread in all organisms. A significant proportion of the transcripts identified in bacteria that have been analyzed using high-throughput methodologies are in antisense orientation, from *E. coli* (26) to *Staphylococcus* (27) or *Prochlorococcus*, a cyanobacterium with a streamlined genome (28). By using a combination of RNA-seq with a previously available TSS data set (7) and a prediction of Rho-independent transcriptional terminators, in this work, we provide a transcriptomic map of *Nostoc* sp. PCC 7120, and show that in this heterocystous cyanobacterium, similar to the observations made for other bacteria, a large number of TUs (65%) overlap another TU in the antisense orientation. We have focused on nitrogen-regulated antisense transcripts as a potential source of regulation in the context of adaptation to nitrogen deficiency and, specifically, in the differentiation of heterocysts.

Two major categories of asRNAs can be considered. On the one hand, mRNAs that are transcribed in a convergent (tail to tail) or, less frequently, in a divergent (head to head) disposition may overlap because their long 5′ or 3′UTRs invade the transcriptional space of adjacent genes. In these cases, the consequences of nitrogen-regulated transcription of a given transcript go beyond the expression of the gene(s) included, potentially affecting the expression of oppositely oriented adjacent genes via antisense transcription (some examples are shown in Fig. S3). In contrast to overlapping mRNAs, a second group of asRNAs, sometimes referred to in the literature as cis-antisense or *bona fide* asRNAs, includes noncoding RNAs transcribed antisense to an mRNA (see examples in Figs. 1, 2, and S4). In this work, we have validated several of these asRNAs that are transcribed from NtcA- or HetR-dependent promoters (Figs. 1 and 2). These observations suggest that the two transcriptional regulators that control both the response to nitrogen limitation and the differentiation of heterocysts could carry out posttranscriptional regulation mediated by nitrogen-regulated asRNAs.

Overlapping asRNAs could be particularly abundant in cyanobacteria as a consequence of the absence of the Rho termination factor, which has been involved in the repression of pervasive, including antisense, transcription (29, 30). Because Rho protein is not found in the cyanobacterial clade (12, 13), transcriptional termination is based on the structure of the nascent mRNA, which usually produces longer 3′UTRs in cyanobacteria (Fig. S1C). We observed a clear preference for 5′UTRs of around 20–40 nucleotides in the four transcriptomes tested (Fig. S1B), which may reflect an optimization for translation (16). The cyanobacterial transcriptomes analyzed also showed longer 5′UTRs, with some 5′UTRs longer than 500 nucleotides (Fig. S1B). Long 5′UTRs may have regulatory functions. Our observation that HetR-regulated genes have longer 5′UTRs than nonregulated genes (Fig. S2) supports this possibility. Long 5′UTRs can also generate overlapping head-to-head mRNAs. For example, the presence of an 800-nt long 5′UTR in *conR* mRNA (TU00137), a gene essential for diazotrophic growth (31), overlaps in a head-to-head disposition with *alr0188* (GDP-mannose pyrophosphorylase). The commonly accepted concept of operon, a genomic arrangement in which genes involved in the same metabolic pathway appear clustered together to facilitate coupled regulation, may be extended to antisense regulation. The genomic arrangement of some overlapping antisense transcripts may also be used for coupled regulation.

The physiological role of antisense transcription remains a matter of debate (32, 33). Pervasive, nonregulated, antisense transcription points to a global function in RNA processing (34) or transcription-coupled DNA repair (35). In contrast, asRNAs whose expression responds to environmental cues and hence becomes differentially expressed under certain conditions or, as exemplified here by heterocyst-specific asRNAs, in a specific cell type, are suggestive of a functional role in the adaptation to certain environmental changes. In both *Listeria monocytogenes* and *Staphylococcus aureus*, a subset of asRNAs are transcribed from SigB-dependent promoters (27, 36), suggesting a role under specific conditions. Here, we describe several asRNAs whose expression responds to nitrogen availability and is regulated in a NtcA- or HetR-dependent manner. In fact, some of the asRNAs described here belong to one of the previously defined groups of HetR-dependent transcripts (6) and exhibit expression profiles that match those of well-known genes involved in heterocyst differentiation and function, again suggesting a functional relevance in the heterocyst differentiation process.

Here, we show that *as_gltA* is HetR dependent and is expressed specifically in heterocysts. Its overexpression reduces the amount of *gltA* mRNA and citrate synthase activity. Therefore, our results suggest that one specific metabolic adaptation of the heterocyst is the reduction of citrate synthase activity, resulting in reduced flow through the tricarboxylic acid (TCA) cycle. We can envision several consequences of such a reduced flow. The two substrates of citrate synthase, oxalacetate and acetyl-CoA would be more available for alternative pathways upon inhibition of this enzymatic activity in the heterocyst. Heterocysts synthesize cyanophycin, an aspartate–arginine polymer (37). Reduction of flow through the TCA cycle would favor the channeling of oxalacetate toward aspartate synthesis. Similarly, a greater pool of acetyl-CoA would be available for heterocyst-specific biosynthetic pathways, including envelope glycolipids (5). In fact, partial repression of citrate synthase activity, resulting in increased Acetyl-CoA pools, has been shown to be a successful strategy to increase carbon partitioning and biofuel production in unicellular cyanobacteria (38). Therefore, the *as_gltA* described here would participate in the metabolic remodeling of heterocysts, similar to the previously described heterocyst-specific asRNA to the *glpX* gene (Fig. S4) that contributes to the shutdown of CO_2_ fixation in heterocysts by negatively regulating sedoheptulose-1,7 bisphosphatase (10). Additionally, a heterocyst-specific asRNA to gene *alr5059* (6) (Fig. S4) could modulate the accumulation of its product, recently described as *ana*Cyp40, a cyclophilin that regulates the assembly of the photosystem and the association of the phycobilisome (20). One particular circumstance that hinders the identification of HetR-dependent, heterocyst-specific RNAs, including asRNAs, is the fact that they are transcribed only in the cells that are differentiating (usually <10% of the cells in the filaments) but not in vegetative cells. In this context, the observation that a heterocyst-specific asRNA appears in our transcriptomic data for whole filaments with a strong change of expression implies very strong induction at the single cell level (i.e. in developing heterocysts). Taken together, these observations suggest that regulatory mechanisms orchestrated by asRNAs may represent a major strategy to regulate metabolic remodeling in heterocysts.

The specific mechanisms used by asRNAs in the regulation of gene expression range from co-degradation of RNA duplexes by RNases to regulation of mRNA traducibility (8, 32, 33). It should be mentioned that the output of antisense interactions might be either positive or negative. In fact, previous work in *Synechocystis* sp. PCC6803 showed that both modes of regulation can be functionally relevant (39–42). We can speculate about the possible regulatory output of the transcription of some of the nitrogen-regulated asRNAs described here. For example, heterocyst-specific transcription of TU03408 (*as_susA*, Fig. S3) could contribute to the down-regulation of *susA expression* in heterocysts, consistent with the observed down-regulation of the expression of the *susA* promoter in these cells (43). Similarly, nitrogen-regulated transcription of TU00968 (*as_all1549-patD*, Fig. S3) could affect the expression of *all1549*, involved in ppGpp metabolism and required for heterocyst differentiation (44). The negative effects of overlapping mRNAs would be consistent with those previously described for *as_furA* (45) or hypothesized for *as_fraE*, which is transcribed as an extension of the *fraF* mRNA, and represents an example of two convergent genes with related functions but having opposite effects (19). This arrangement represents a paradigm in RNA-mediated regulation, the excludon concept (46). Dedicated RNAs could also constitute a mechanism to regulate one particular gene in an operon. As an example, one of the validated NtcA-regulated asRNAs (TU03089, *as_pstS2*, Fig. 1) could regulate an ABC transporter for phosphate whose four subunits (PstB2, PstA2, PstC2, and PstS2) are cotranscribed in a single mRNA (TU03088). While transcription of this mRNA shows little variation upon nitrogen removal, transcription of *as_pstS2*, which overlaps only the first gene in the transcript, encoding the substrate binding subunit, is strongly induced upon nitrogen removal, suggesting that this asRNA could regulate a constitutively expressed transporter by affecting only the expression of the subunit involved in substrate recognition.

The morphological and metabolic transformation of certain cells of a cyanobacterial filament into a heterocyst requires different gene expression programs in adjacent cells. One mechanism behind such expression patterns is the occurrence of heterocyst-specific promoters, which have long been described for most genes involved in differentiation (4, 7), and also contributes to the proper transcription of housekeeping genes whose expression patterns must be modulated during differentiation, such as *sigA*, encoding the major sigma factor (47). In this context, the existence of noncoding transcripts, both sRNAs and asRNAs, with cell-specific expression could also contribute to the onset of cell-specific expression programs.

In this work, we have shown that there are several asRNAs with heterocyst-specific regulated expression that could be involved in modulating gene expression in heterocysts. In fact, we demonstrate that an antisense to *gltA*, which is strongly induced in developing heterocysts, regulates citrate synthase expression. asRNAs constitute an additional layer of regulation that seems to be involved in heterocyst differentiation. Further study of the catalog of heterocyst-specific asRNAs described here would reveal details on how heterocysts achieve differential gene expression beyond transcriptional regulation by protein factors.

## Materials and methods

### Strains and growth conditions

The different *Nostoc* sp. PCC 7120 strains used in this work (Table S1) were grown in BG11 medium (48) as detailed in Supplementary Materials and Methods. *Escherichia coli* strains (Table S1) were grown in LB medium, supplemented with appropriate antibiotics (49).

### Construction of *Nostoc* sp. PCC 7120 derivative strains

Details on the construction of plasmids (Tables S2 and S3) and strains are given in the Supplementary Materials and Methods.

### RNA preparation, library processing, and RNA-seq analysis

Total RNA was isolated using hot phenol as described (50) with modifications (51). RNA samples were treated with turbo DNase (Invitrogen), and strand-specific libraries were prepared using Illumina Stranded TOTAL RNA preparation RIBO-ZERO PLUS kit and sequenced on the Illumina platform NextSeq500 at the Genomics Core Facility of Cabimer (Seville, Spain). Raw RNA-Seq data can be accessed in the GEO database under accession number GSE212705. Sequencing reads were mapped with HISAT2 (52) and the prediction of TUs was carried out by ANNOgesic (17). Fragments associated with TUs were counted using HTseq (53) and differential expression analysis was carried out using *edgeR* (54) and *limma* (55) package in R. Previously published data were used for the analysis of TUs in *Synechocystis* sp. PCC 6803 (56), *C. jejuni* 81116 (14), and *H. pylori* 26695 (15, 16).

Further details about the bioinformatic methods are given in the Supplementary Materials and Methods.

### Northern blot analysis, 3′RACE and primer extension assays

Strand-specific ^32^P probe labeling and Northern blots were performed as detailed in the Supplementary Materials and Methods. The RNA used as a probe for *as_gltA* was transcribed in vitro (MEGAscript Kit; Life Technologies, #AM1330) from PCR-generated DNA templates using the primers indicated in the Table S2. 3′RACE (10) and primer extension analysis (57) were performed as previously described using the oligonucleotides specified in Table S2.

### Fluorescence microscopy

Details of fluorescence quantification are given in the Supplementary Materials and Methods.

### Citrate synthase assay

The crude extracts were prepared in 50 mM Tris-HCl pH 8 buffer by mechanically disrupting the cells using glass beads. Protein concentration was determined using the Bradford method (58) and citrate synthase activity was determined using a 5′,5′-dithiobis-(2-nitrobenzoate) colorimetric assay (59). Details about the composition of the reaction mixtures are given in the Supplementary Materials and Methods.

### Statistical methods

The Wilcoxon–Mann–Whitney test and Student's t test were used to determine statistical significance. The use of each test and the number of biological samples can be found in the figure legends.

## Supplementary Material

pgad187_Supplementary_Data

## Data Availability

All data are included in the manuscript and/or supporting information. Raw RNA-Seq data can be accessed in the GEO database under accession number GSE212705.
