# Supplementary material for: Nitrogen-regulated antisense transcription in the adaptation to nitrogen deficiency in Nostoc sp. PCC 7120
Source: PNAS Nexus. 2023 Jun 2;2(6):pgad187. doi: 10.1093/pnasnexus/pgad187 (PMC10287535; doi:10.1093/pnasnexus/pgad187)
Supplement: pgad187_Supplementary_Data [file pgad187_supplementary_data.zip › PNASNEXUS-PNASNEXUS-2023-00150R-s01.pdf]

PNAS nexus

Supplementary Information for

Nitrogen-regulated antisense transcription in the adaptation to nitrogen deficiency in *Nostoc* sp. PCC 7120

Manuel Brenes-Álvarez, Agustín Vioque, and Alicia M. Muro-Pastor*

*Alicia M. Muro Pastor
Email: alicia@ibvf.csic.es

This PDF file includes:

- Supplementary text
- Figures S1 to S4
- Tables S1 to S3
- Legend for Dataset S1
- SI References

Other supplementary materials for this manuscript include the following:

- Dataset S1

Supplementary Information Text

Supplementary Material and Methods

Strains and growth conditions. Cultures of *Nostoc* sp. PCC 7120 wild type, *ntcA* mutant (1) and *hetR* mutant 216 (2) were bubbled with an CO_2/air mixture (1% v/v) and grown photoautotrophically at 30ºC in BG11 medium (3) containing ferric citrate instead of ammonium ferric citrate, lacking $NaNO_3$ but containing 6 mM NH_4Cl, 10 mM $NaHCO_3$, and 12 mM N-tris (hydroxymethyl) methyl-2-aminoethanesulfonic acid-NaOH buffer (pH 7.5). Nitrogen deficiency was induced by filtering, washing and resuspending cells in nitrogen-free BG11 medium containing 10 mM $NaHCO_3$. For the culture of overexpressor strains, cells were incubated with agitation at 30ºC for 1 week in flasks containing liquid BG11 medium and nitrogen deficiency was induced as described above, resuspending cells in nitrogen-free medium. Culture media were solidified with 1% Bacto Agar (Difco). Strains used in this work are described in Table S1. *Nostoc* strains bearing plasmids pMBA51, pMBA75, pMBA104, pMBA105, or pMBA117 were grown in the presence of streptomycin (Sm) and spectinomycin (Sp), 2 µg/ml each (liquid medium) or 3 µg/ml each (solid medium). *E. coli* strains were grown in LB medium, supplemented with appropriate antibiotics (4).

RNA preparation, library processing, and RNA-seq analysis. Total RNA was isolated using hot phenol as described (5) with modifications (6) and treated with Turbo DNase (Invitrogen) to eliminate DNA traces. Strand-specific libraries compatible with Illumina sequencing were prepared using Illumina Stranded TOTAL RNA preparation RIBO-ZERO PLUS kit at the Genomics Core Facility of Cabimer (Seville, Spain) and sequenced on the Illumina platform NextSeq500.
Sequencing raw data consisted of 2 fastq files comprising 17 to 21 million paired-end reads for each sample. Quality control was carried out using FASTQC (https://www.bioinformatics.babraham.ac.uk/projects/fastqc/). The *Nostoc* sp. PCC 7120 Refseq genome (sequence and annotation from chromosome and plasmids) was retrieved from NCBI (https://www.ncbi.nlm.nih.gov/genome/13531?genome_assembly_id=300961) and sequencing reads were mapped with HISAT2 (Kim 2019) using default parameters and "-no-spliced-alignment" parameter.
We have modified the annotation of the available Refseq genome for *Nostoc* sp. PCC7120 (May 2022), as follows. Instead of using "systematic_names", we used "old_locus_tag" as gene names. Whenever a systematic name corresponded to an old locus tag, the latter was used. The remaining systematic names without a correspondence to the previous annotation of the genome were kept as such. We have incorporated additional available information into the annotation file. Genes with a gene name assigned in the literature were renamed according to available

information. In addition, we changed gene names according to the homologies predicted by UNIPROT (https://www.uniprot.org/).

Conventional fragmented RNA-seq libraries from this study were combined with a dataset obtained from dRNA-seq libraries (7) to predict transcriptional units (TU). TUs were predicted using ANNOgesic (8) considering previously determined TSS (7) and a prediction of transcriptional terminators carried out by transtermHP (9), using the "terminator" function of ANNOgesic. We used default parameters for the prediction of terminators and TU. Only transcripts with an average coverage higher than 30 reads and predicted in the two biological replicates were considered. No overlapping transcripts in the same strand (subtranscripts) were allowed and previous dRNA-seq data were used to assign a TSS to a transcript. The transcript annotation generated by ANNOgesic was later processed and manually curated as follows. If the start or end of a putative TU was closer than 50 nucleotides to a TSS or terminator, the transcript was extended to the position of the TSS or terminator, respectively. Some predicted TUs were merged or split into several TUs due to their regulation or the existence of experimental data previously reported in the literature. Antisense TUs with no TSS assigned and an average coverage lower than 50 reads were discarded. Finally, sRNAs previously reported to be expressed in the conditions used in this study were also included as a transcript in case they were not predicted by ANNOgesic because of their small size.

We used HTSeq (10) with "-m union" parameter to count the number of fragments associated to each TU. The output of HTseq was processed using *edgeR* (11) and *limma* (12) packages in R. CPM (counts per million reads) was used as the normalization method to counteract different library sequencing depth per sample. We performed the following comparisons WT_9h vs. WT_0h, WT_24h vs. WT_0h, and WT_24h vs. WT_9h. TU with an absolute $\log_2$(foldchange)>1 and a p-value < 0.05 were considered statistically differentially expressed features. To visualize the RNA-seq coverage along the genome, BAM files were split by strands using SAMtools (13). Bamcoverage (14) was later used to obtain the number of counted fragments per nucleotide along the whole genome.

Raw RNA-Seq data can be accessed in the GEO database under accession number GSE212705.

Comparison between transcriptomes of different organisms. The length distribution of transcripts, 5' UTRs and 3'UTRs of *Synechocystis* sp. PCC 6803 was obtained directly from (15). The transcriptomes of *Campylobacter jejuni* 81116 and *Helicobacter pylori* 26695 were assembled using ANNOgesic and previously published transcriptomic data (16-18). The dRNA-seq data for these organisms were retrieved from NCBI GEO under accession numbers GSE38883 and GSE67564, respectively. Conventional RNA-seq data from *Helicobacter pylori* 26695 was also retrieved from NCBI SRA under accession number SRR031126. The parameters

for TSS prediction were optimized using “optimized” function from ANNOgesic, TSS were predicted using dRNA-seq data, and TU prediction was carried out using ANNOgesic with default parameters. The statistical significance of different length distribution of transcripts, 5’UTRs and 3’UTRs was determined using the Wilcoxon-Mann-Whitney test.

Northern blot analysis, 3’ RACE and primer extension assays. The oligonucleotides used are described in Table S2. RNA for Northern blot hybridization of *as_glt*A was separated in 8% urea-polyacrylamide as described (19) (7 µg of total RNA). Samples for Northern blot hybridization of *gltA* mRNA were treated with DNase (Turbo DNA free kit, Invitrogen), separated in 1% agarose denaturing formaldehyde gels, and transferred to Hybond-N+ membrane (GE Healthcare) using 20X SSC buffer.

The membrane in Fig. 3*C* was hybridized with a single-stranded *as_gltA* radioactively labelled RNA probe transcribed *in vitro* from a PCR-generated template as described (19) (see Table S2 for oligonucleotides). Oligonucleotide 964 was end-labeled with [γ-^{32}P] ATP and polynucleotide kinase and used as a probe for *as_gltA* (Fig. 4*B*), while a one-strand probe for *gltA* (Fig. 4*C*) was labeled using oligonucleotide 1050 using a PCR product (oligonucleotides 1049+1050) as template. Hybridization to 5S rRNA or *rnpB* (20) was used as a loading and transfer control.

3′-RACE assays were carried out essentially as described (21), with 6 µg of dephosphorylated total RNA (rAPid Alkaline phosphatase, Roche). The oligonucleotides used are listed in Table S2. The 3'RNA-Adapter (50 pmol) was ligated to the *Nostoc* sp. PCC 7120 total RNA at 25°C for 2 h with T4 RNA ligase (New England Biolabs). After phenol/chloroform extraction and ethanol precipitation, the RNA was reverse-transcribed with Superscript III (Invitrogen; 200 U) using 10 pmol of primer 281, complementary to the 3'RNA-adapter. The reverse transcription products were amplified by PCR using MyTaq DNA Polymerase (Bioline) with primers 282 (nested to 281) and gene-specific primer #933 using 1 µl of reverse transcription reaction as template. The PCR products were cloned in the pMBL-T cloning vector (Canvax Biotech) and 10 clones were sequenced. Primer extension analysis of 5´ ends was performed as previously described (22) using the oligonucleotides described in Table S2 labeled with [γ-^{32}P] ATP.

Construction of *Nostoc* sp. PCC 7120 derivative strains. We have used pMBA37 (21) as the backbone for overexpressing *as_gltA* or a sponge of *as_gltA*. The sequences encoding *as_gltA* or the sponge of *as_gltA* were amplified from genomic DNA using oligonucleotides 934 and 935 or 942 and 943, respectively (see Table S2 for oligonucleotides). The products were verified by sequencing, digested with NsiI and XhoI, and cloned between the NsiI and XhoI sites in pMBA37 (between the *trc* promoter and the T1 terminator of the *E. coli rrnB* gene), rendering pMBA104 and pMBA105, respectively (Table S3). pMBA51, a plasmid that overexpresses a control RNA corresponding only to the T1 terminator under the *trc* promoter (21), pMBA104 and pMBA105

were introduced in *Nostoc* wild type by conjugation as described (23) generating strains OE_C, OE_as_gltA and OE_as_as_gltA (sponge of *as_gltA*), respectively (Table S1).
To generate a plasmid that contains a fusion between the promoter of *gltA* or *as_gltA* and a promoterless *gfpmut2* gene in *Nostoc*, the promoter regions were amplified from genomic DNA using oligonucleotides 976 and 977 or 753 and 754, respectively. The products were checked by sequencing, digested with ClaI-XhoI, and cloned in ClaI-XhoI digested pSAM270 (24) rendering pMBA117 and pMBA75, respectively. pMBA117 and pMBA75 were introduced in *Nostoc* wild type by conjugation (23).

Fluorescence microscopy. Fluorescence images of *Nostoc* sp. PCC 7120 filaments carrying plasmid pMBA75 or pMBA117 subjected to nitrogen deficiency on plates were taken four days after plating in media without combined nitrogen using an Olympus FLUOVIEW FV3000 confocal laser-scanning microscope equipped with an UPlanApo 60x / 1.5 NA oil immersion objective. Samples were excited at 488 nm by an argon ion laser and fluorescent emission was monitored by collection across windows of 500–540 nm (GFP) and 650–750 nm (cyanobacterial autofluorescence). The accumulation of GFP was quantified using Olympus software and the images were further processed with ImageJ (25) for visualization purposes.

Citrate synthase assay. Cells from 50 ml cultures were harvested by filtration, washed with 50 mM Tris-HCl pH 8, and resuspended in 500 µl of the same buffer containing the EDTA-free protease inhibitor cocktail (Roche). Cell suspension was mixed with glass beads (SIGMA, 200 µm) in an Eppendorf tube and subjected to 10 cycles of 1 min vortexing plus 1 min of cooling on ice. The crude extract was separated from the cell debris and the unbroken cells by centrifugation (10 min at 17000 x *g* at 4 °C) and used for the citrate synthase activity assay. Protein concentration was determined using the Bradford method (26). Citrate synthase activity was determined in the crude extracts using a 5',5'-dithiobis-(2-nitrobenzoate) (DTNB) colorimetric assay (27) . Reaction mixtures contained 25 mM Tris-HCl pH 7.5, 20 mM $MgCl_2$, 1 mM oxalacetate, 0,1 mM acetyl-CoA, 0,1 mM DTNB and 0.3-0.6 µg of crude extract. The increase in absorbance at 412 nm was monitored at 30ºC in 96-wells microtiter plates using a VarioskanLux (Thermo Fisher Scientific).

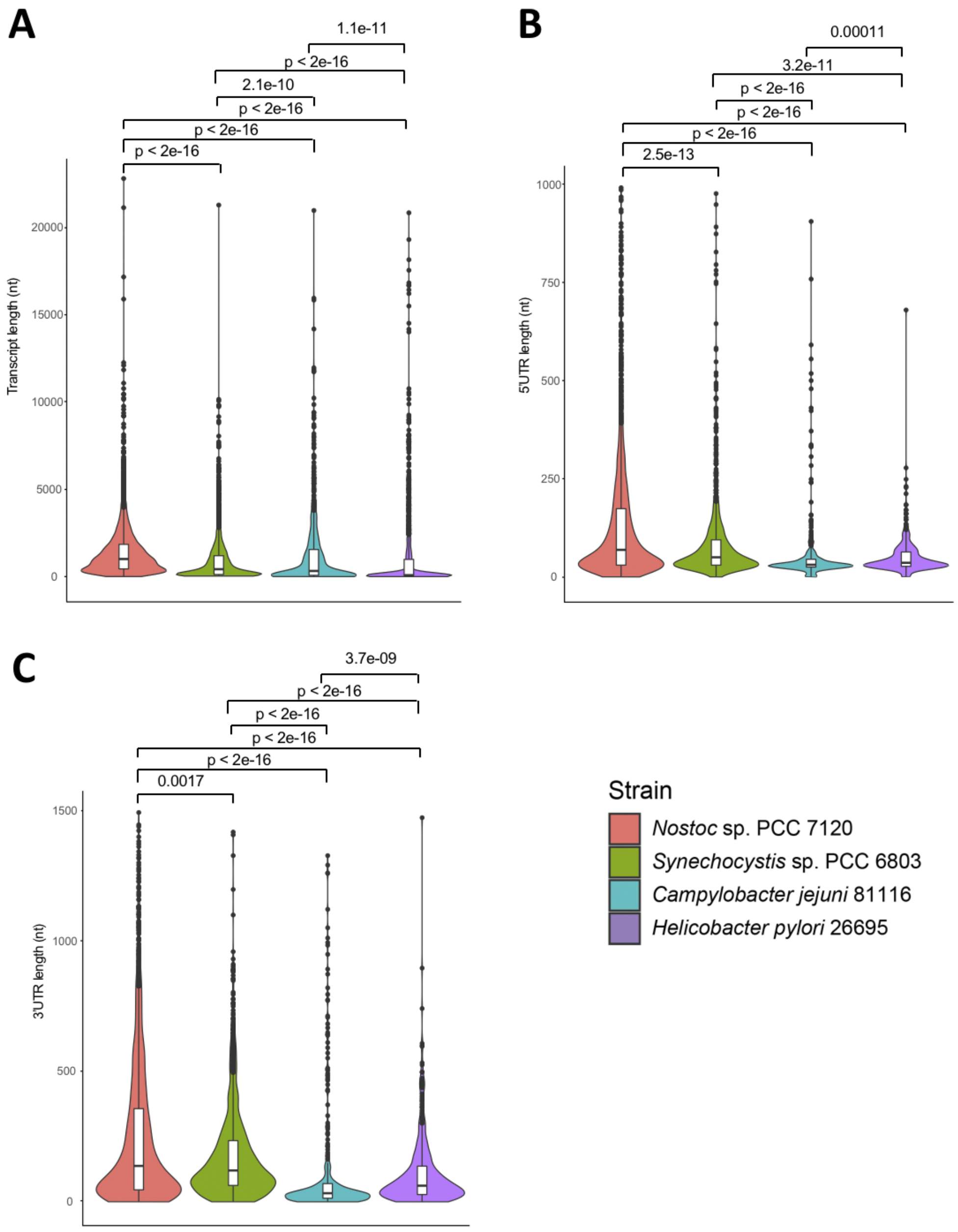

Fig. S1. Comparison of the size distribution of transcripts length, 5'UTRs length and 3'UTRs length between *Nostoc* sp. PCC 7120 and other bacteria. Violin and boxplots of the size distribution of transcript length (A), 5'UTR length (B), and 3'UTR length (C) are shown for *Nostoc* sp. PCC 7120 (this work), *Synechocystis* sp. PCC 6803 (15), *Campylobacter jejuni* (16), and *Helicobacter pylori* (17, 18). The adjusted p-values for statistical significance according to the Wilcoxon-Mann-Whitney test are also shown for each comparison.

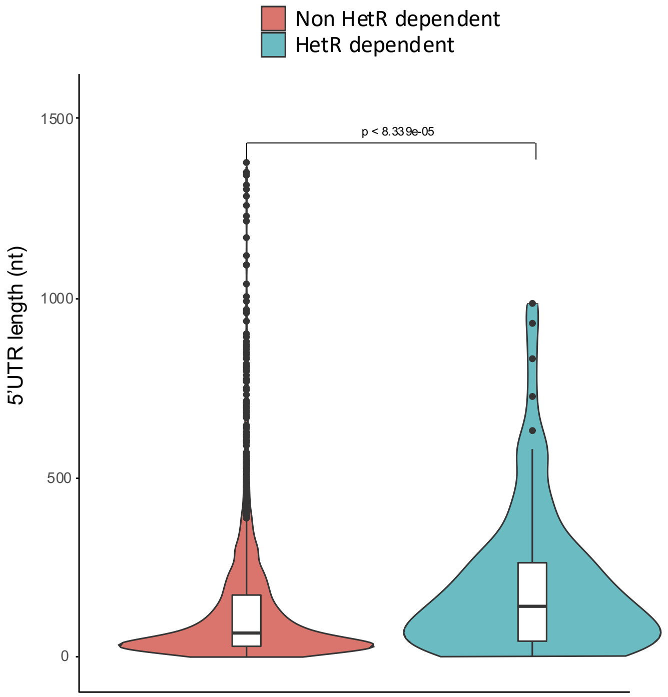

Fig. S2. Comparison of the size distribution of 5'UTRs length between transcriptional units that contain HetR-dependent genes and all other transcriptional units. A total of 94 transcriptional units containing protein coding genes classified as HetR-dependent in a previous clustering analysis (clusters early-DIF and late-DIF) (24) were manually tabulated. The length of their 5'UTRs was compared with the 5'UTR length in all the other protein coding transcriptional units. The adjusted p-value for statistical significance according to the Wilcoxon-Mann-Whitney test is also shown.

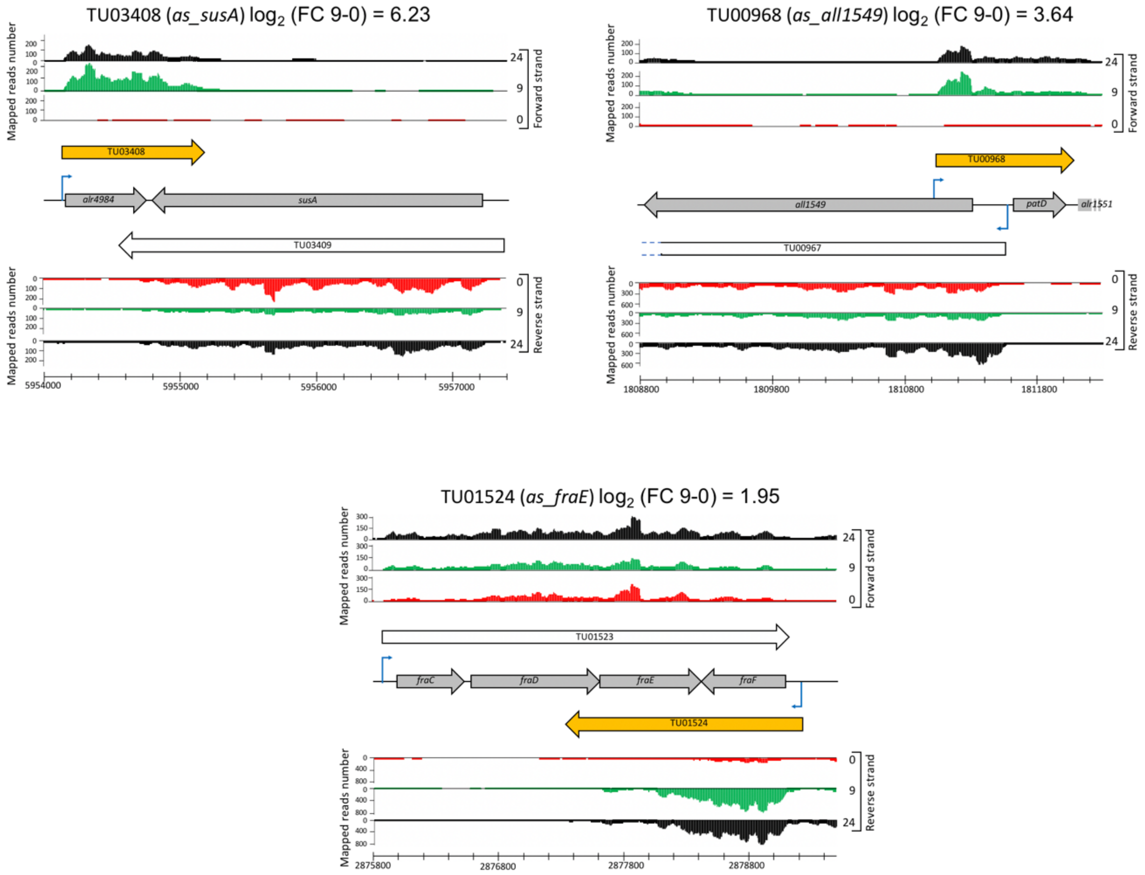

Fig. S3. Examples of mRNA-mRNA antisense genomic arrangements involving one nitrogen-regulated mRNA. The mapped reads distribution of RNA obtained from cells grown in the presence of NH_4^+ (red), or after 9 (green) or 24 h (black) in the absence of combined nitrogen. Genomic regions containing as_*susA*, as_all1549, and *as_fraE* are shown. Annotated ORFs are represented by gray arrows. TUs are represented by white arrows with their corresponding identification. Nitrogen-regulated antisense TUs are colored orange. Previously identified TSS are indicated by bent blue arrows. Genomic coordinates denote the position of the *Nostoc* sp. PCC 7120 chromosome. The scale indicates the number of reads mapped per nucleotide position.

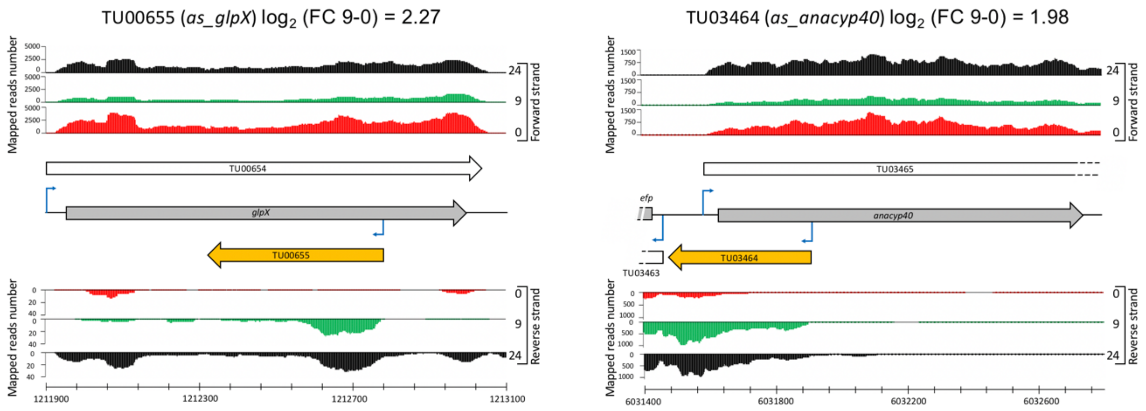

Fig. S4. Previously described nitrogen-regulated non-coding asRNAs. The mapped reads distribution of RNA obtained from cells grown in the presence of NH_4^+ (red), or after 9 (green) or 24 h (black) in the absence of combined nitrogen. Genomic regions containing *as_glpX* and *as_anacyp40* are shown. Annotated ORFs are represented by gray arrows. TUs are represented by white arrows with their corresponding identification. Nitrogen-regulated antisense TUs are colored orange. Previously identified TSS are indicated by bent blue arrows. Genomic coordinates denote the position in the *Nostoc* sp. PCC 7120 chromosome. The scale indicates the number of reads mapped per nucleotide position.

Table S1. Strains.

Strain	Description	Reference
Escherichia coli		
DH5α	Used for routine transformation	(28)
***Nostoc* sp.**		
PCC 7120	Wild type	Pasteur Culture Collection
CSE2	*ntcA* mutant	(1)
216	*hetR* mutant (S179N)	(2)
OE_C	$Sm^R Sp^R$, pMBA51 inserted into plasmid alpha. *T1* terminator of *E. coli rrnB* gene expressed constitutively from the *trc* promoter.	(21)
OE_as_gltA	$Sm^R Sp^R$, pMBA104 inserted into plasmid alpha. *as_gltA constitutively* transcribed from the *trc* promoter.<br>Overexpression of *as_gltA*	This work
OE_as_as_gltA	$Sm^R Sp^R$, pMBA105 inserted in plasmid alpha. Sponge of *as_gltA* expressed constitutively from the *trc* promoter.<br>Overexpression of a sponge of *as_gltA*.	This work
P_{gltA}::GFP	$Ap^R Sm^R Sp^R$, pMB117 inserted into plasmid alpha. *sfGFPmut2* under the control of the *gltA* promoter	This work
P_{as_gltA}::GFP	$Ap^R Sm^R Sp^R$, pMBA75 inserted in plasmid alpha. *sfGFPmut2* under the control of the *as_gltA* promoter	This work

Table S2. Oligonucleotides.

Name	Sequence (5’-3’)	Used for
387	CTCCTAAAGTCCCCACAGCG	Template for *in vitro* transcription of an RNA probe for *as_gltA*
797	**TAATACGACTCACTATAGGGTTCGGG**ATGCTGT AGTGCGGC	
479	GAATTCGTAATTCGTTATACCC	Primer extension *as_hglD*
753	GTTTTATCGATTAGCAACCACCGCATACGGG	Construction of a P_{as_gltA}-*gfpmut2* fusion
754	GTTTTCTCGAGAGATTATTCCGCCAATTTTCTC	
931	CCGTCGAGATTTGCACAATCC	Primer extension *as_gltA*
3’ RNA adapter	[Phos]UUCACUGUUCUUAGCGGCCGCAUGCUC-IdT	3’ RACE
281	GAGCATGCGGCCGCTAAG	
282	GGCCGCTAAGAACAGTG	
933	TAACGGGGTCATTACCTTTCC	
934	GTTTTATGCATTGGCGGAATAATCTAAATCATC	Construction of pMBA104
935	GTTTTCTCGAGATTAAATACCGGATTCGGGATAT	
942	GTTTTATGCATATTAAATACCGGATTCGGGATAT	Construction of pMBA105
943	GTTTTCTCGAGTGGCGGAATAATCTAAATCATC	
964	CAATGGATGCACTCCAAGCCTCTGCGGCGGCTT TAGGC	Probe for *as_gltA*
965	TCAAGGAATTATCCCGCAAGC	Primer extension *as_acsF*
966	TCCTGTGTTGGATTTGAAAGC	Primer extension *as_fdxB*
968	TGTTGCTGACCAAATCAGTCC	Primer extension *as_pstS2*
969	TTACCTCACGAAGTTAGCCAG	Primer extension *as_leuA*
970	CAAACAGGAGAACTCATTTGG	Primer extension *as_pknC*
976	GTTTTATCGATGCTAGAAGAATCAAATCATCTTGC	Construction of a P_{gltA}-*gfpmut2* fusion
977	GTTTTCTCGAGAGAACATCTGACTACGACCCAC	
1049	GCATACGATGAATGCTTCCAC	Probe for *gltA* mRNA
1050	AGGAATACCCATCTTCCGATAC	

Restriction sites used for cloning (underlined) and T7 promoter (bold) are indicated.

Table S3. Plasmids.

Name	Description	Reference
pSAM270	$Ap^RSm^RSp^R$, plasmid containing a fusion of P_{hetR} to *gfpmut2* used as the backbone for the integration in alpha plasmid of ClaI-XhoI digested fragments containing transcriptional fusions of promoters to *gfpmut2*.	(24)
pMBA37	$Ap^RSm^RSp^R$, plasmid for the overexpression of transcripts from the *trc* promoter and followed by the *T1* terminator of the *E. coli rrnB* gene, used as transcriptional terminator.	(21)
pMBA51	$Ap^RSm^RSp^R$, control plasmid, expresses a 56 nt transcript derived from the *T1* terminator of the *E. coli rrnB* gene from the *trc* promoter.	(21)
pMBA75	$Ap^RSm^RSp^R$, PCR fragment generated with primers 753 and 754 cloned as a ClaI-XhoI fragment in pSAM270 to generate a fusion of P_{as_gltA} to *gfpmut2*.	This work
pMBA104	$Ap^RSm^RSp^R$, plasmid based on pMBA37 for the overexpression of *as_gltA* from the *trc* promoter.	This work
pMBA105	$Ap^RSm^RSp^R$, plasmid based on pMBA37 for the overexpression of a sponge of *as_gltA* from the *trc* promoter.	This work
pMBA117	$Ap^RSm^RSp^R$, PCR fragment generated with primers 976 and 977 cloned as a ClaI-XhoI fragment in pSAM270 to generate a fusion of P_{gltA} to *gfpmut2*.	This work

Dataset S1 (separate file). Transcriptional units predicted in this work. For each transcriptional unit (TU) the assigned number, start, end, length, strand, and replicon are indicated. The TSS previously determined (7) associated with TUs are also shown. Genes and locus names are indicated. Genes and loci in brackets correspond to genes not fully covered by TUs. TUs in antisense disposition to other TUs (with at least 50 overlapping nucleotides) are also shown. The 5'UTR and 3'UTR lengths are calculated based on the distance between the start and end of the TUs to their fully covered associated gene. Sheet 2 shows information about nitrogen-regulated TUs, including information about log_2-foldchange and adjusted p-value for comparisons analyzed in this work.

SI References

1. J. E. Frías, E. Flores, A. Herrero, Requirement of the regulatory protein NtcA for the expression of nitrogen assimilation and heterocyst development genes in the cyanobacterium *Anabaena* sp. PCC 7120. *Mol. Microbiol.* **14**, 823-832 (1994).
2. W. J. Buikema, R. Haselkorn, Characterization of a gene controlling heterocyst differentiation in the cyanobacterium *Anabaena* 7120. *Genes and Dev* **5**, 321-330 (1991).
3. R. Rippka, J. Deruelles, J. B. Waterbury, M. Herdman, R. Y. Stanier, Generic assignments, strain stories and properties of pure cultures of cyanobacteria. *J. Gen. Microbiol.* **111**, 1-61 (1979).
4. J. F. Sambrook, D. W. Russell, "Molecular cloning: A laboratory manual". (Cold Spring Harbor Laboratory, Cold Spring Harbor, N Y, 2001).
5. A. Mohamed, C. Jansson, Influence of light on accumulation of photosynthesis-specific transcripts in the cyanobacterium *Synechocystis* 6803. *Plant Mol. Biol.* **13**, 693-700 (1989).
6. M. Brenes-Álvarez, E. Olmedo-Verd, A. Vioque, A. M. Muro-Pastor, Identification of conserved and potentially regulatory small RNAs in heterocystous cyanobacteria. *Front. Microbiol.* **7**, 48 (2016).
7. J. Mitschke, A. Vioque, F. Haas, W. R. Hess, A. M. Muro-Pastor, Dynamics of transcriptional start site selection during nitrogen stress-induced cell differentiation in *Anabaena* sp. PCC7120. *Proc. Natl. Acad. Sci. USA* **108**, 20130-20135 (2011).
8. S. H. Yu, J. Vogel, K. U. Forstner, ANNOgesic: a Swiss army knife for the RNA-seq based annotation of bacterial/archaeal genomes. *GigaScience* **7**, 1-11 (2018).
9. C. L. Kingsford, K. Ayanbule, S. L. Salzberg, Rapid, accurate, computational discovery of Rho-independent transcription terminators illuminates their relationship to DNA uptake. *Genome Biol.* **8**, R22 (2007).
10. S. Anders, P. T. Pyl, W. Huber, HTSeq--a Python framework to work with high-throughput sequencing data. *Bioinformatics* **31**, 166-169 (2015).
11. M. D. Robinson, D. J. McCarthy, G. K. Smyth, edgeR: a Bioconductor package for differential expression analysis of digital gene expression data. *Bioinformatics* **26**, 139-140 (2010).
12. M. E. Ritchie *et al.*, *limma* powers differential expression analyses for RNA-sequencing and microarray studies. *Nucleic Acids Res.* **43**, e47 (2015).
13. H. Li *et al.*, The Sequence Alignment/Map format and SAMtools. *Bioinformatics* **25**, 2078-2079 (2009).
14. F. Ramírez *et al.*, deepTools2: a next generation web server for deep-sequencing data analysis. *Nucleic Acids Res.* **44**, W160-165 (2016).
15. M. Kopf *et al.*, Comparative analysis of the primary transcriptome of *Synechocystis* sp. PCC 6803. *DNA Res.* **21**, 527-539 (2014).
16. G. Dugar *et al.*, High-resolution transcriptome maps reveal strain-specific regulatory features of multiple *Campylobacter jejuni* isolates. *PLoS Genet.* **9**, e1003495 (2013).
17. T. Bischler, H. S. Tan, K. Nieselt, C. M. Sharma, Differential RNA-seq (dRNA-seq) for annotation of transcriptional start sites and small RNAs in *Helicobacter pylori*. *Methods* **86**, 89-101 (2015).
18. C. M. Sharma *et al.*, The primary transcriptome of the major human pathogen *Helicobacter pylori*. *Nature* **464**, 250-255 (2010).
19. C. Steglich *et al.*, The challenge of regulation in a minimal photoautotroph: non-coding RNAs in *Prochlorococcus*. *PLoS Genet.* **4**, e1000173 (2008).
20. A. Vioque, Analysis of the gene encoding the RNA subunit of ribonuclease P from cyanobacteria. *Nucleic Acids Res.* **20**, 6331-6337 (1992).
21. E. Olmedo-Verd, M. Brenes-Álvarez, A. Vioque, A. M. Muro-Pastor, A heterocyst-specific antisense RNA contributes to metabolic reprogramming in *Nostoc* sp. PCC 7120. *Plant Cell Physiol.* **60**, 1646-1655 (2019).

22. A. M. Muro-Pastor, A. Valladares, E. Flores, A. Herrero, The *hetC* gene is a direct target of the NtcA transcriptional regulator in cyanobacterial heterocyst development. *J. Bacteriol.* **181**, 6664-6669 (1999).
23. J. Elhai, C. P. Wolk, Conjugal transfer of DNA to cyanobacteria. *Methods Enzymol* **167**, 747-754 (1988).
24. M. Brenes-Álvarez *et al.*, Elements of the heterocyst-specific transcriptome unravelled by co-expression analysis in *Nostoc* sp. PCC 7120. *Environ. Microbiol.* **21**, 2544-2558 (2019).
25. J. Schindelin *et al.*, Fiji: an open-source platform for biological-image analysis. *Nat. Methods* **9**, 676-682 (2012).
26. M. M. Bradford, A rapid and sensitive method for the quantitation of microgram quantities of protein utilizing the principle of protein-dye binding. *Anal. Biochem.* **72**, 248-254 (1976).
27. P. A. Srere, Citrate synthase. *Methods Enzymol.* **13**, 3-11 (1969).
28. D. Hanahan, Studies on transformation of *Escherichia coli* with plasmids. *J. Mol. Biol.* **166**, 557-580 (1983).
